# Economic evaluations of non-communicable disease interventions in developing countries: a critical review of the evidence base

**DOI:** 10.1186/1478-7547-4-7

**Published:** 2006-04-03

**Authors:** Jo-Ann Mulligan, Damian Walker, Julia Fox-Rushby

**Affiliations:** 1 Health Economics and Financing Programme, London School of Hygiene and Tropical Medicine, UK; 2 Bloomberg School of Public Health, Department of International Health, Johns Hopkins University, USA; 3 Health Economics Research Group, Brunel University, UK

## Abstract

**Background:**

Demographic projections suggest a major increase in non-communicable disease (NCD) mortality over the next two decades in developing countries. In a climate of scarce resources, policy-makers need to know which interventions represent value for money. The prohibitive cost of performing multiple economic evaluations has generated interest in transferring the results of studies from one setting to another. This paper aims to bridge the gap in the current literature by critically evaluating the available published data on economic evaluations of NCD interventions in developing countries.

**Methods:**

We identified and reviewed the methodological quality of 32 economic evaluations of NCD interventions in developing countries. Developing countries were defined according to the World Bank classification for low- and lower middle-income countries. We defined NCDs as the 12 categories listed in the 1993 World Bank report *Investing in Health*. English language literature was searched for the period January 1984 and January 2003 inclusive in Medline, Science Citation Index, HealthStar, NHS Economic Evaluation Database and Embase using medical subheading terms and free text searches. We then assessed the quality of studies according to a set of pre-defined technical criteria.

**Results:**

We found that the quality of studies was poor and resource allocation decisions made by local and global policy-makers on the basis of this evidence could be misleading. Furthermore we have identified some clear gaps in the literature, particularly around injuries and strategies for tackling the consequences of the emerging tobacco epidemic.

**Conclusion:**

In the face of poor evidence the role of so-called generalised cost-effectiveness analyses has an important role to play in aiding public health decision-making at the global level. Further research is needed to investigates the causes of variation among cost, effects and cost-effectiveness data within and between settings. Such analyses still need to take a broad view, present data in a transparent manner and take account of local constraints.

## Background

The next two decades are predicted to see dramatic changes in the health needs of the developing world. Whilst developing nations are still struggling with the unfinished agenda of communicable diseases (in particular HIV/AIDS, malaria and tuberculosis) a major increase in NCD mortality is predicted, as depression, heart disease and cancer replace infectious diseases as the leading causes of disability and premature death [1]. The steep projected increase in the burden of NCDs worldwide is not only driven by demographic changes, but also by the rapidly increasing numbers of people who smoke or who are exposed to other risks such as obesity, physical inactivity and heavy alcohol consumption. Factors including rapid urbanisation and industrialisation are implicated in the increase in neuropsychiatric disorders such as depression and alcohol dependence.

Undoubtedly, estimates of the current and future burden of disease have stimulated the recognition of the importance of NCDs [2]. However, while information on the burden of disease can illustrate the magnitude of a disease, it provides no guidance in terms of how best to deal with it [3,4]. If decisions are to be made about where to direct scarce resources, policy-makers need to know which of the available interventions to tackle these diseases are the most efficient and equitable.

In 2000 WHO re-emphasised the role that cost-effectiveness analysis can have in identifying the interventions able to provide most health gain from available resources [5]. Cost-effectiveness analyses have also been included in sectoral allocation exercises for countries seeking health sector loans from the World Bank. However, these have occurred only in a limited number of countries and with a limited number of interventions [6]. Hence there is an urgent need to establish which interventions are cost-effective in developing country settings. Given the expense of such exercises, the capacity to undertake analyses and the role of international and bi-lateral agencies in funding the evaluation and provision of interventions, there is also much interest in being able to generalise the models and findings from economic evaluations across developing countries.

Given the increasing importance of economic evaluation as a decision-making tool, it is imperative that the quality of evaluations undertaken is monitored. Two reviews of the quality of studies aimed at parasitic and communicable diseases found that few published economic evaluations had been performed, diseases and geographical areas had been neglected and for the most part the quality of studies was poor [7,8]. However, the quality of existing evaluations of NCD interventions in developing countries has never been systematically evaluated, hence very little is known about the existing evidence base. Although Jamison et al. reviewed the available literature on NCDs in 1993, no systematic attempt was made to evaluate the methodological quality of the studies [9].

This paper has three objectives: to critically appraise approaches taken in costing and the assessment of outcomes, as well as methods of analysis and interpretation; to consider the quality of the evidence concerning the efficiency of NCD interventions and its potential contribution to decision-making; and to examine the applicability of best practice in economic evaluation of NCDs in developing country settings.

## Methods

The review sought to identify published economic evaluations of interventions aimed at NCDs in developing countries. Developing countries were defined according to the World Bank classification for low- and lower middle-income as having a gross national income of less than $9,205 per capita. We defined NCDs as the 12 categories listed in the 1993 World Bank report *Investing in Health *(cancer, cardiovascular, diabetes, neuropsychiatric, nutritional, oral health, digestive diseases, respiratory, sense organ, congenital abnormalities, genitourinary, muskoskeletal) plus unintentional and intentional injuries [10]. English language literature was searched for the period January 1984 and January 2003 inclusive, in Medline, Science Citation Index, HealthStar, NHS Economic Evaluation Database and Embase using medical subheading terms and free text searches (See Table 1). We also used reviews of economic evaluations of NCD interventions [9-11]. Finally, we contacted experts in the field for further references. Since this was a review of the published literature we did not search the 'grey' literature.

**Table 1:** Search strategy

1 Medline, HealthStar, PreMedline and PubMed
Thesaurus **cost-benefit analysis **[all subheadings] **combined with **thesaurus: Malignant Neoplasms, Cancer, Diabetes, Nutritional and endocrine, Neuropsychiatric, Sense organ, Cardiovascular, Respiratory, Digestive, Genitourinary, Musculoskeletal, Congenital abnormalities, Oral health, Injuries.
2 Medline, HealthStar, NEED, King's Fund Library database, Science Citation Index (SCZZ), Social Science Citation Index (SSCI), Embase (EMZZ)
Text searches: cost* and benefit*; cost* and effect*; and cost* and utility* **in combination **with the above diseases

Our first search yielded 273 items. After reviewing the titles and abstracts to reject any from high-income countries, 61 articles appeared to match our inclusion criteria. These papers were obtained and reviewed but nearly half (n = 29) were subsequently excluded because they were: review articles, editorials or letters (n = 15); cost analyses with no details on effects (n = 7); or cost-outcome analyses and hence not full economic evaluations (n = 7) [12].

A set of review questions was developed based on Drummond et al.'s checklist which covered background questions (e.g. country, disease, intervention) and technical characteristics (perspective adopted, covering aspects of cost, outcomes and types of analysis/interpretation) [12].

## Results

### Background characteristics

We identified 32 articles published in 19 journals between the years 1984 and 2003 [13-44]. Most articles were published in a country or regional based journal, e.g. *East African Medical Journal *and the *South African Medical Journal *rather than more general journals such as *The Lancet*. Cost-effectiveness studies were the main study type (n = 23) followed by cost minimisation (n = 8), cost utility (n = 1) and cost-benefit analyses (n = 1). It is worth noting that although several papers described themselves as cost-benefit analyses, they were in fact cost-effectiveness studies because health benefits had not been valued in monetary units. Table 2 shows that the majority of studies were carried out in Asia (n = 16) and sub-Saharan Africa (n = 14) with only five considering the Americas.

**Table 2:** Comparison between the number of papers published by region and regional estimates of the burden of non-communicable disease

Region	Number of papers*	%	Burden of disease (hundreds of thousands of DALYs lost)**	%
Sub-Saharan Africa	14	44%	1050	18%
Asia and other Islands	11	34%	1040	18%
Latin America and the Caribbean	4	13%	689	12%
India	3	9%	1714	29%
China	2	6%	1431	24%
Total	34	100	5924	100

* Some papers performed multiple analyses and/or used multiple sources of data etc. Therefore, the presentation of results may suggest that there are more than 32 papers, which is not the case.

**Source: World Development Report 1993 [10]

Table 3 shows that the papers covered a broad range of disease groups. The most common focus was nutritional disorders (n = 8) followed by half the number of papers on cardiovascular and neuropsychiatric diseases. Table 3 also shows that the majority of studies concentrated on treatment (n = 23) rather than prevention (n = 8) or diagnosis (n = 7). Within this classification there was a range of interventions. For example, interventions with a treatment focus included the evaluation of a new drug or a surgical technique (n = 9) and the management of a disease such as diabetes or epilepsy in different settings (n = 6). Of the eight studies which examined preventive strategies, three examined nutritional interventions to prevent vitamin deficiency, two studies examined the impact of anti-smoking strategies (both as part of a broader package of interventions) and one study looked at legislation to prevent road traffic injuries. Other examples included a school based dental programme and a home-visiting scheme for psychiatric patients. The seven studies with a diagnostic focus included cancer screening interventions (n = 3), and studies which examined the diagnosis of sickle cell anaemia and renal failure (n = 2).

**Table 3:** Study focus

Focus	Diagnosis	Prevention	Treatment	Number of papers*
Nutritional	1	3	6	8
Cardiovascular		1	4	4
Neuropsychiatric	1	1	3	4
Cancer	3	1	1	3
Injury/Trauma		1	3	3
Digestive		1	2	2
Genitourinary	1		1	2
Respiratory			2	2
Sense organ			2	2
Congenital abnormalities	1			1
Diabetes			1	1
Muskoskeletal		1		1
Oral health		1		1
Other surgical			1	1
Number of Papers*	7	8	23	

* Some papers performed multiple analyses. Therefore, the presentation of results may suggest that there are more than 32 papers, which is not the case. For this reason the sum of rows and columns does not always equal the number of papers.

It was not possible to determine the source of funding for eight studies. Where it was possible to deduce the source of funding, public sponsorship was overwhelmingly the most common (n = 23). Among public funding bodies were national governments (n = 15); international organisations such as the World Health Organization (WHO) and the World Bank (n = 3) and non-governmental organisations or charitable foundations (n = 2). Private support from a pharmaceutical company was stated in one paper.

### Technical characteristics

#### Perspective

Relatively few authors stated the perspective of their paper explicitly. However it was possible to infer the perspective of all papers after examining the costs included. The health care provider was the perspective most often used (n = 25). One study took the perspective of both the provider and patients, and one study examined patient costs only. Any study that also included indirect costs or benefits was defined loosely as having taken a societal perspective and, using this definition, five studies took this perspective.

#### Costs

With one exception,[23] all studies in the review included some direct health care provider costs, although the scope of these varied enormously. Often authors simply included the costs of the intervention(s) under study and assumed all other costs (such as overheads) to be the same. Other studies were more comprehensive and included overhead costs and capital costs. Occasionally the total cost of an intervention was cited but it was impossible to determine what was included in the estimate. Three papers explicitly considered donated inputs. For the five studies that took a societal perspective, indirect costs consisted of wage losses due to morbidity and mortality and time spent seeking treatment.

Many studies used actual expenditure data (n = 17) as their source of data. Seven studies used published sources to generate cost estimates sometimes supplemented with expert opinion. Two studies used expert opinion only. Sometimes, however, it was not possible to see exactly how costs were derived (n = 6).

The majority of studies (n = 17) appeared to use an 'ingredients' costing approach where costs were broken down between the main cost components such as vehicles, salaries and consumables. Fewer (n = 5) used an 'activity' based approach, by identifying specific tasks such as programme and therapy costs. Two studies appeared to use some combination of the two and it was not possible to discern the approach for eight papers as only the final total costs were provided. Regardless of the approach taken, most papers (n = 21) presented aggregated cost information so it was impossible to ascertain whether all the previously identified costs had been measured. Only seven studies provided details of resources consumed in physical units, such as numbers of staff employed or time spent on a particular activity.

#### Valuation of costs

The costs of donated inputs, such as people's time, was generally valued using unskilled or minimum wage rates. All of the studies used market prices of one form or another to value the costs of interventions but none discussed the possibility that these prices may not fully reflect the opportunity cost of resources. Currencies included: US dollars (n = 12); UK pounds (n = 1) with the remaining studies using local currencies (n = 16). In addition, three studies quoted costs in both US dollars and the local currency. The possibility that exchange rates may not always reflect the true economic costs and benefits of importing and exporting was addressed by one study, which noted the potential impact of local import duties on equipment costs [21].

#### Consequences

Primary data were generated by epidemiological studies, including experimental (i.e. randomised controlled trials) and quasi-experimental (i.e. intervention versus control but not randomised, or before-and-after) (n = 14) and observational studies (n = 9). Studies using secondary data (n = 9) generally used published data from other studies and generated results using decision tree analysis or other basic modelling techniques such as applying a published mortality rate to the study population.

Papers presented a range of outcome measures ranging from impact on process to final outcomes. Process (e.g. length of stay, hospitalisation rate) or intermediate case-specific outcome measures (e.g. number of cases of cancer detected) were presented in every paper. The most frequently presented final outcome measures were impact of an intervention on morbidity e.g. cases prevented (n = 23) and mortality e.g. deaths averted or life years gained (n = 8). Only one study used a measure of effects combining quantity and quality of life (disability adjusted life years (DALYs)), the calculation of which was based on local (Nepalese) life expectancies, utility values derived in Canada, and unpublished studies on visual acuity following treatment [29]. Finally, one study expressed outcomes in terms of monetary units, by estimating the impact of correcting iron deficiencies on the productivity of agricultural workers [26].

#### Types of analyses and interpretation of results

The most frequently used summary measure for cost-effectiveness and cost-utility analyses was average cost-effectiveness ratios (n = 20). Three studies also examined the additional costs and benefits associated with shifting to a competing alternative using incremental analysis to estimate incremental cost-effectiveness ratios. As cost-minimisation analyses compare interventions that have the same effect, the eight cost-minimisation analyses in the review used either least cost or net savings as their summary measure. The cost-benefit analysis used a benefit/cost ratio as its summary measure.

The mean duration of interventions studied was 1.6 years (range: 1 month – 5 years) and whilst the mean analytic horizon was longer at 3.6 years (range: 1 month – 33 years), the majority of papers (n = 18) analysed costs and effects for one year or less. It was impossible to ascertain the time horizons for three papers. Given that the majority of studies adopted an annual time horizon, discounting was effectively ignored by most papers. However, of the 11 papers with differential timing, only three papers used a discount rate; two papers used a rate of 3 per cent and one used a rate of 10 per cent.

Less than half (n = 14) of the papers undertook any kind of sensitivity analysis to assess the robustness of their findings to assumptions about input parameters or model structure. Of these, one-way sensitivity analysis was the most common technique (n = 12). Two studies used threshold analysis and one performed a multi-way analysis. None considered the structure of the model. Some studies (n = 5) provided reasons for the choice of sensitivity analysis. For instance, one study stated that there were uncertainties about most of the values used to calculate the cost-effectiveness ratios [27]. Another indicated that data in the baseline analysis that had to be estimated was subsequently tested using sensitivity analysis [25]. A further study examined the impact of bias introduced by non-randomisation of the study group [33]. More often, however, studies stated that a sensitivity analysis was undertaken to confirm the 'robustness of the results' but justifications for the particular methods of analysis or the choice of variables to alter was not always clear.

Only four studies (13% of papers) explicitly mentioned the likely impact of implementing the intervention on the annual total health budget. Another eight mentioned issues of affordability but only in very brief terms, for example by noting simply that the available budget should be taken into account (but without providing any details), or by questioning the sustainability of a novel service such as a mobile cervical cytology screening service where there are already established health services [16].

The costs and effects of interventions and their alternatives can potentially vary greatly from one context to another. The extent to which the results or models from economic evaluations are generalisable to other settings is thus important to consider. Although many papers (n = 13) made an attempt to address this issue, efforts were largely confined to simply stating the limitations of the study, such as whether randomisation was employed or noting one or two facts about the study site which might limit generalisability to other contexts. One paper argued that the result for the country in question (Nepal) might be more expensive than other countries because of high transportation costs due to the difficult terrain [27]. Another study from Sri Lanka argued that the presence of higher socio-economic groups and greater private medical insurance coverage might limit the generalisability of their results [31]. Overall, of the 13 papers that mentioned generalisability issues, five stated that results were not generalisable to other settings while only one explicitly claimed that the methods and interpretation should be generalisable to other settings [22].

## Discussion

We could identify only 33 economic evaluations aimed at NCDs published between 1984 and 2003 with 26 of these published in the years following the 1993 World Development Report. It is roughly equivalent to one study per four developing countries and is less than a quarter of the number found in a similar review for communicable diseases [7]. However, we do recognise the following limitations: the search was confined to English language publications only; and it excluded unpublished and 'grey' literature domain. On the other hand, we wanted to capture material that was likely to be relatively easily accessible to decision-makers in developing countries.

The above notwithstanding, we believe our findings represent an alarming paucity of evidence and reflect the concerns of others in the field that there is a gap in NCD research in developing countries. Mendis et al suggest that "fragile research capacity, inadequate financial investment, langauge barriers and exclusions of journals edited in developing countries in MEDLINE are some of the factors that probably contribute to this situation" [45]. We are inclined to agree with this view.

Viewing the proportions of papers alone in relation to the burden of disease suggest that injuries are particularly under-represented relative to nutritional disorders (see Table 4) and that, geographically, sub-Saharan Africa and Asia are over-represented relative to India and China in terms of the number of studies published (see Table 1). However, this takes no account of the expenditure on interventions in these areas and the disease categories mask gaps in specific intervention areas. For example, only two studies [17,42] looked at anti-smoking interventions and in both cases these interventions were not the main focus of the analysis. This is despite predictions of an emerging tobacco epidemic influencing both cardiovascular diseases and cancer. Similarly, we could find only three studies aimed at injury or trauma [21,22,36] and only one of these [22] looked at prevention rather than treatment. Overall, it seems that the existing evidence base from developing countries is very unlikely to be able to challenge effectively any resource allocation decisions made on the basis of burden of disease estimates without a great deal more investment in the number of economic evaluations across countries and health interventions.

**Table 4:** Study focus and burden of disease

	Proportion of papers with focus	Proportion of NCD DALYs in low and middle income countries, 1990*
Nutritional	23%	6%
Cardiovascular	11%	16%
Neuropsychiatric	11%	18%
Cancer	9%	9%
Injury/Trauma	9%	28%
Digestive diseases	6%	5%
Genitourinary	6%	2%
Respiratory	6%	7%
Sense organ	6%	2%
Congenital abnormalities	3%	3%
Diabetes	3%	1%
Muskoskeletal	3%	2%
Oral health	3%	1%

* Source: World Health Report 1999 [57] (Annex Table 3). Expressed as a proportion of DALYs attributable to: NCDs, nutritional deficiencies and injuries

The studies reviewed here raise a further question concerning the extent to which we might expect differences in the number of studies aimed at non-communicable diseases compared to communicable diseases in developing countries. There are several reasons why this might be the case. First, inevitably, focus at the national and international level remains on the unfinished agenda of communicable disease. Second, many NCD interventions such as cancer prevention and screening are complex and currently still unaffordable in developing countries. Equally, many cost-effective NCD interventions emphasise life style and preventive interventions, but these tend to be aimed at older people. Arguably this group are not given as high a priority by funders of evaluations compared to the target group of many communicable disease interventions: children and pregnant women. These reasons go some way to explain the paucity of data and emphasise why there is perhaps a greater need to increase the methodological rigour of the few studies that are undertaken in this area.

However, to simply invest in increasing the number of economic evaluations requires careful consideration as our review has highlighted a number of concerns with the existing evidence base that resonate with the findings of other reviews [7,46-48]. At times studies are opaque, miss opportunities to facilitate decision-making and give almost no consideration to the generalisability of results or models to other countries. We explain our reasoning for each below along with the implications.

### Too many studies are opaque

The basic tasks of any economic evaluation are to identify, measure, value and compare the costs and consequences of the alternatives being considered. Ideally, utilisation data should be presented alongside average cost data in order for readers to see how total costs are constructed [49]. However the lack of transparency in many of the studies meant it was impossible to ascertain what authors had actually done. For example, six studies did not cite the source of cost data and in many studies price and quantity data were aggregated so it was not always clear whether all appropriate costs were measured and valued. In others it was not clear whether the full range of costs were considered because inadequate descriptions of the range of alternatives were provided. Relatively few studies considered the possibility of costs falling on patients and their caregivers, even though these costs are potentially important and their inclusion could change overall recommendations [50].

The main implication of these findings is that the internal validity of many papers could not be judged. Should decision-makers therefore not use such results? Whilst we may want to conclude 'yes', given the paucity of data it seems unlikely that there are many good alternatives and it may be better to have partially informed estimates or adjusted estimates than no estimates at all.

### Few findings are interrogated rigorously for their own setting

We found little critical examination of findings in relation to the study site. Over half of papers undertook no sensitivity analysis at all. In reality, however, every evaluation will contain some degree of uncertainty or imprecision. Differences in the perspective of an analysis, alternative methodological approaches or assumptions used to derive estimates of costs and outcomes, may have dramatic effects on the results. Of those that did undertake sensitivity analysis, simple one-way analysis was the main method used, with only two studies developing this to a threshold analysis. Whilst one-way sensitivity analysis is helpful in understanding the impact of assumptions about individual variables, by itself it is an under-estimate of how uncertain the estimated overall cost-effectiveness ratio actually is. Various forms of multi-way sensitivity analysis offer a relatively easy route to a qualitative exploration of the uncertainty concerning parameter inputs [51]. with statistical analyses available for calculating confidence intervals/ellipsoids [52,53]. Finally, it was also notable that no paper questioned or examined the impact of the structure behind, or process of developing, the implicit or explicit models of cost-effectiveness.

### Opportunities to facilitate decision-making are often lost

A critical choice in the application of economic analyses to aid resource allocation is the choice of outcome measure. All studies used some form of process or intermediate case-specific units as a measure of effect. While this is acceptable for assessing technical efficiency (which is concerned with how best to meet a given objective at least cost) it does not help in choosing how to allocate resources across different programmes with different outcomes because like cannot be compared with like. It does not facilitate a sectoral perspective to be taken where the costs and effectiveness of all possible interventions are compared in order to select the mix that maximises health or some other objective function for a given set of resource constraints. This problem is compounded given the lack or transparency of reporting the source of data and disaggregated effects, as other evaluators are unable to make the link between disease averted and gains in health or welfare. If, as predicted, the burden of NCDs rises in relation to the already considerable burden of communicable diseases then choices will need to be made between options across the disease spectrum.

Thus the challenge is for economic evaluations to use outcome measures that facilitate comparisons across the health sector. Deaths averted or life years gained, presented in 25% of papers, were the main outcome measures comparable across diseases and health interventions. However, as NCDs rise in relation to communicable disease, measuring only deaths averted may underestimate health gain from interventions to prevent or treat NCD as a significant impact may be realised in terms of quality of life and not only quantity of life.

The scant attention paid to issues of affordability and sustainability are also worrying. In particular in developing countries, where significant proportions of inputs may be imported or donated, it is important to estimate the current and potential future financial costs. Very few contrasted the economic costs of introducing a new programme on overall health expenditure or considered separately the cost of introducing and running a new programme.

### Generalisability

Ultimately, a good study should help the user interpret the results for use in other settings. However, the majority of papers avoid considering the generalisability of their results and models or simply state that their results either are, or are not, relevant to other settings or countries but produce no supporting evidence. Many studies also adopted an incremental costing approach from which it is more difficult to transfer/generalise findings, unless the prior level of existing services and infrastructure is clearly specified, which was rarely the case.

At a basic level, consideration of the generalisability of results would be improved if cost data was disaggregated by price and utilisation level, to allow other analysts to adapt results to their own circumstances. Alternatively an investigation of how and why costs and effects vary within the study site would allow some judgement about the impact of independent variables on cost, effects and cost-effectiveness in another setting. Table 5 is adapted from Jamison et al [54]. and provides some examples of the factors influencing variation in cost-effectiveness of NCD interventions. We suggest that researchers may find such variables a useful starting point for contemplating the design and analysis of future economic evaluations.

**Table 5:** Factors influencing variation in cost-effectiveness

**Influencing factor**	**Examples**
*Epidemiological environment*	
Prevalence of condition	Screening and referral programs for breast cancer
Incidence of condition	Preventive measures for many injuries
Existence of competing risks of synergisms	Some surgical interventions: among the very young or elderly, competing risks reduce the cost-effectiveness of some targeted interventions
*Individual characteristics*	
Age	Cancer treatment: more cost-effective for younger patients
Tendency to compliance	Anti-hypertensive medication
Tendency to self-refer	Diabetes control
Level of risk factors	Hypertension and hyperlipdemia
Individual variation in values	Attitude toward disability relative to risk of death; can lead to individual differences in intervention effectiveness
*System characteristics*	
Local costs of non-traded inputs to health care system	Real costs of care intensive interventions (such as hospitalisation after trauma) are low where wages are low, because most health care personnel are relatively immobile
Generalised systemic competence	Cost-effectiveness at the margin of some interventions in a system with a low level of professionalism and capacity may be much higher than in more developed systems
Discount rate	Where discount rates are high, interventions with payoffs well into the future (such as treatment of obesity) become relatively less attractive.

Source: adapted from Table 1–4 in Jamison [54].

Given the paucity of evidence, the need to make decisions and the likelihood of the leagues tables of cost-effectiveness of health interventions in many regions of the world, it is imperative that researchers explore issues to do with generalisability within their own analysis. However, analysts have expressed concern that studies may be too context specific and that a great deal of effort would need to be spent on incorporating detailed information on local costs, social concerns and political constraints for the results/models to be generalised to another setting [55]. The response to the problem of poor quality context specific economic evaluations has been the arrival of the generalized cost-effectiveness analysis (CEA) [56]. Yet the emergence of league tables showing the cost-effectiveness of interventions for different regions has raised further questions. What evidence is used to generate such results? How are primary data be collected and if so by whom? What is the role of existing data? And how applicable are data from NCD economic evaluations conducted in developed countries to developing countries? Only a very small number of economic evaluations have been undertaken on NCDs in developing countries in recent years and, as we have shown, much of it is of dubious quality and limited in scope. Decisions made on the basis of the existing evidence base could result in the inefficient use of scarce resources. Furthermore, given the apparent preoccupation with the evaluation of communicable disease interventions in developing countries, the case for ensuring best practice in economic evaluations of NCDs is given added urgency. This is not to say that the methods for conducting economic evaluations of NCDs are any different from those applied to communicable disease. Rather, we believe there is 'added' value from ensuring that future economic evaluations undertaken in developing country settings are conducted in such a way as to improve their internal and external validity.

If, in the absence of appropriate evidence, results from developed countries are applied to developing country settings via generalised cost-effectiveness analyses then there needs to be a good understanding of which factors have the most impact on the results. In the context of NCDs, several parameters that have a large influence on the results of studies might differ substantially between developed and developing countries. For example in the case of cancer, this includes the age-specific incidence of cancer, all cause life expectancy, the age structure of the population, cancer treatment effectiveness and the cost in the absence of the preventive or screening intervention.

The clear advantage of the generalised cost-effectiveness approach is that it can be presented in a way that can be translated across different settings [56]. However it is also acknowledged that 'global or regional cost-effectiveness results may have limited relevance for local settings and policy processes'. A way through this is via the 'contextulisation' of generalized CEA to the country level. This is achieved in a number of ways from assuming interventions are done in a technically efficient way through to modifying analyses to capture some local variations such as differences in the use of labour [56]. Because the evidence base for the economic evaluation of NCDs is so weak, generalised CEA will have an increasingly important role to play in public health decision making, so long as local factors are fully taken into account.

## Conclusion

What are the implications for future economic evaluations in developing countries? First, future economic evaluations need to take a broader view and present data in a transparent way. We have also identified some clear gaps in the literature, particularly around injuries and strategies for tackling the consequences of the emerging tobacco epidemic in developing countries. Interventions to control chronic diseases such as diabetes mellitus and hypertension are also under-represented. Second, more research is needed which investigates the causes of variation among cost, effects and cost-effectiveness data within and between settings. Once this has been done, a third priority is to undertake more work on assessing the transferability/generalisability of existing and future evaluations within and between settings and the results need to be explicitly tested in different settings.

## Competing interests

The author(s) declare that there are no competing interests

## Authors' contributions

DW and JFR conceived the idea of the study. JM designed the data extraction tool, undertook the review and wrote the first draft of the manuscript. All authors contributed to subsequent redrafting of the manuscript and approved the final version.
